# Cardiogenic shock in takotsubo syndrome: etiology and treatment

**DOI:** 10.1007/s12928-024-01031-3

**Published:** 2024-07-22

**Authors:** Ken Kato, Davide Di Vece, Mari Kitagawa, Kayo Yamamoto, Shuhei Aoki, Hiroki Goto, Hideki Kitahara, Yoshio Kobayashi, Christian Templin

**Affiliations:** 1 Department of Cardiovascular Medicine, Chiba University Graduate School of Medicine, 1-8-1 Inohana, Chuo-ku, Chiba, Japan; 2 Internal Medicine B, University Medicine Greifswald, Greifswald, Germany; 3 First Clinic of Internal Medicine, Department of Internal Medicine, University of Genoa, Genoa, Italy; 4 Center for Molecular Cardiology, Schlieren Campus, University of Zurich, Zurich, Switzerland; 5 Swiss Cardiovascular Clinic, Private Hospital Bethanien, Zurich, Switzerland

**Keywords:** Takotsubo syndrome, Cardiogenic shock, Left ventricular outflow tract obstruction, Acute mitral regurgitation

## Abstract

Takotsubo syndrome (TTS) can mimic acute coronary syndrome despite being a distinct disease. While typically benign, TTS can lead to serious complications like cardiogenic shock. Cardiogenic shock occurs in 1–20% of TTS cases. Various mechanisms can cause shock, including pump failure, right ventricular involvement, left ventricular outflow tract obstruction, and acute mitral regurgitation. Because treatment depends on the mechanism, early identification of the mechanism developing cardiogenic shock is essential for optimal treatment and improved outcomes in TTS patients with cardiogenic shock. This review summarizes current knowledge on causes and treatment of cardiogenic shock in patients with TTS.

## Introduction

Takotsubo syndrome (TTS) is characterized by transient myocardial systolic dysfunction involving the left ventricle (LV) and/or the right ventricle (RV). Clinically, differentiating TTS from acute coronary syndrome (ACS) is crucial as their initial presentations, including symptoms, electrocardiographic changes, and biomarker profiles, are remarkably similar. Although TTS has been recognized as a relatively benign disease mainly because of reversible nature, recent studies demonstrated that serious cardiac complications could occur in the acute phase and TTS patients had comparable in-hospital outcomes with ACS patients [1]. In particular, cardiogenic shock represents one of the important causes of in-hospital mortality.

Given the typical spontaneous recovery of ventricular systolic dysfunction, in-hospital management for TTS is primarily supportive. Because different mechanisms can be related to cardiogenic shock in patients with TTS, early identification of patients at high risk of cardiogenic shock and careful assessment for the etiology of cardiogenic shock are crucial.

This review summarizes current knowledge on etiology and treatment for cardiogenic shock in patients with TTS.

## Prevalence and predictors of cardiogenic shock in TTS

Cardiogenic shock is a major contributor to acute mortality in TTS, with reported incidence ranging from 1% to 20% [2–7]. Di Vece et al. reported from the International Takotsubo Registry (InterTAK Registry) that 9.5% of TTS patients experienced cardiogenic shock during the acute phase [2]. This study also demonstrated that apical type, preceding physical stress, left ventricular ejection fraction (LVEF) < 45%, diabetes mellitus, and atrial fibrillation were independently associated with cardiogenic shock in patients with TTS [2]. In addition, patients with cardiogenic shock showed worse short- and long-term outcomes in comparison to those without cardiogenic shock. A report from the RETAKO Registry demonstrated that 11.4% of TTS patients had cardiogenic shock, and male sex, QTc interval prolongation, lower left ventricular ejection fraction at admission, physical triggers, and presence of significant left ventricular outflow tract pressure gradient were independent predictors of the development of cardiogenic shock [4]. Notably, the RETAKO Registry also found that TTS patients with cardiogenic shock who received β-blockers at discharge had lower 1-year mortality, suggesting a potential benefit for this therapy.

## Etiology and treatment of cardiogenic shock in TTS

**Fig. 1:**
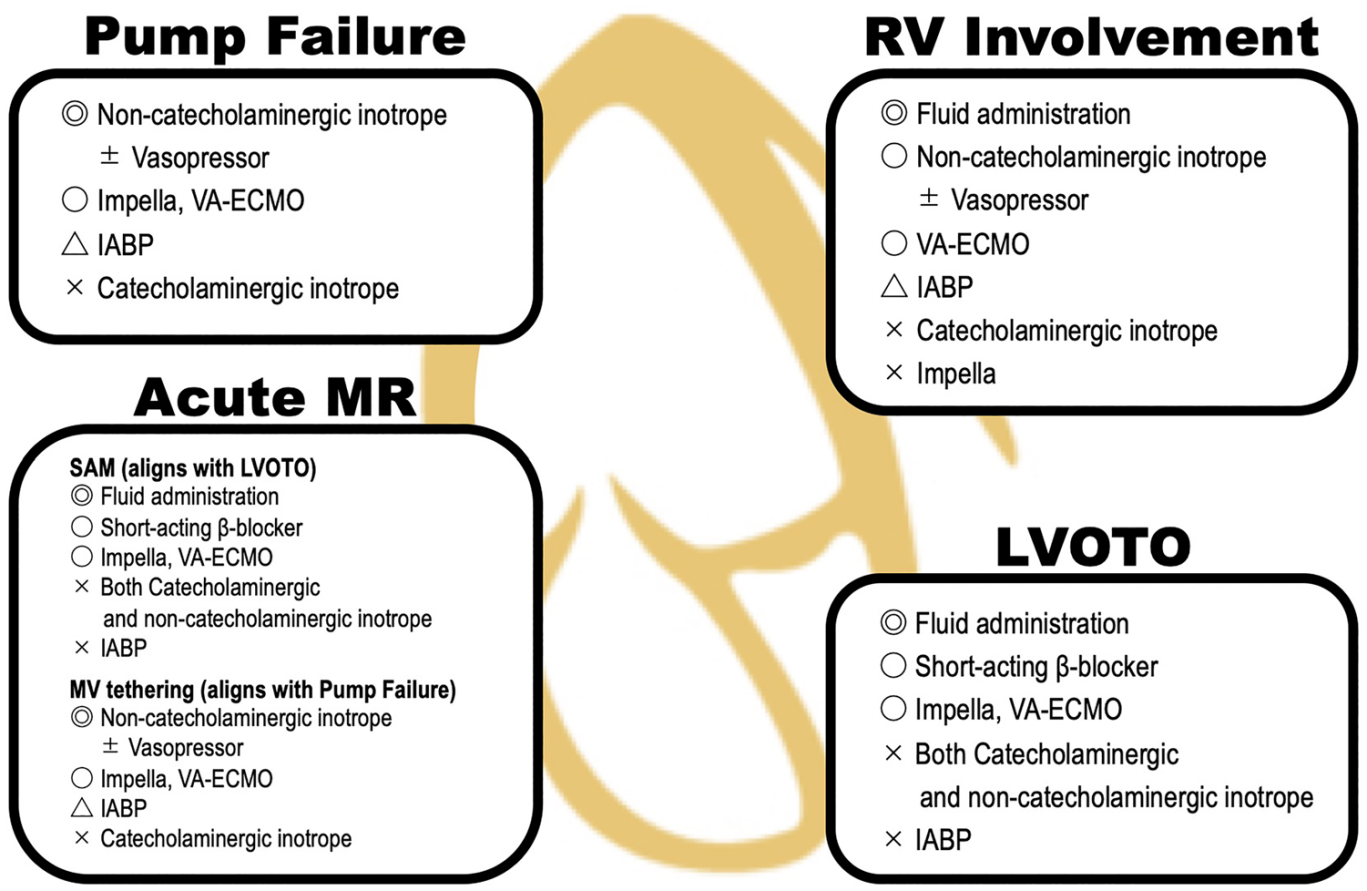
Etiology and management of cardiogenic shock in takotsubo syndrome. *IABP* intra-aortic balloon pump; *LVOTO* left ventricular outflow tract obstruction; *MR* mitral regurgitation; *RV* right ventricular; *VA-ECMO* venoarterial extracorporeal membrane oxygenation

In the acute phase of TTS, various mechanisms can contribute to cardiogenic shock, including pump failure (e.g., extensive left ventricular wall motion abnormality), right ventricular (RV) involvement, left ventricular outflow tract obstruction (LVOTO), and acute mitral regurgitation (MR), often occurring in combination. Identifying the predominant mechanism underlying cardiogenic shock is crucial for selecting the most effective treatment strategy (Fig. 1).

1. Pump failure (Fig. 2)

**Fig. 2:**
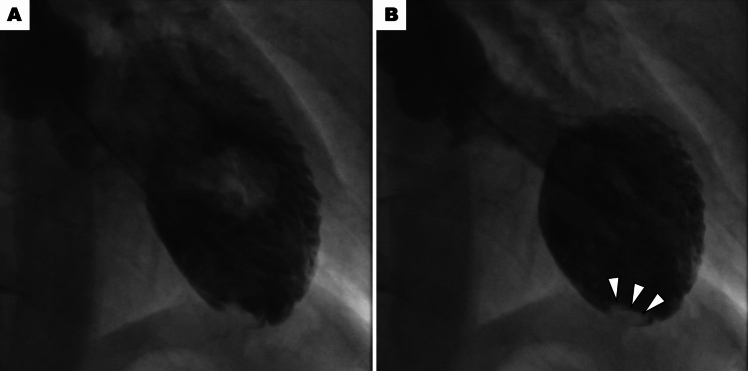
Pump failure. Left ventriculography demonstrates extensive left ventricular wall motion abnormality and low ejection fraction, which is associated with an increased risk of ventricular thrombus formation (white arrows). Panels **A** (diastole) and **B** (systole)
  Extensive LV wall motion abnormalities can lead to cardiogenic shock in TTS due to pump failure. In these cases, LVEF typically falls below 35%. Large-scale multicenter registries demonstrated a strong correlation between lower LVEF and the development of cardiogenic shock [2, 4]. When pump failure due to low LVEF is confirmed as the primary cause (after ruling out LVOTO and RV involvement), the goal of therapy is to improve LV contractility. While catecholaminergic inotropic agents such as dopamine and dobutamine are commonly used in the treatment of heart failure due to low cardiac function in general, experts advise against them in TTS due to a potential link between catecholamines and TTS development [8–11]. As an alternative to catecholamines, the use of non-catecholaminergic inotropic agents such as levosimendan and milrinone might be considered [12, 13]. It is important to note that these agents can also have vasodilatory effects, potentially worsening hypotension. In such cases, a combined approach with vasopressors like norepinephrine (a catecholaminergic agent with high α1 receptor selectivity) and vasopressin (a non-catecholaminergic option) might be necessary.
  If hemodynamic stabilization is not achieved with these medications, mechanical circulatory support may be a valuable option as a bridge to recovery. As intra-aortic balloon pump (IABP) can exacerbate LVOTO in TTS, it should not be used in cases with LVOTO [13, 14]. If IABP is used in cases without LVOTO, close monitoring with echocardiography is crucial to detect any IABP-induced LVOTO development. Recently, Santoro et al. reported from the GEIST Registry that 20% of TTS patients with cardiogenic shock received IABP, and the use of IABP was not associated with lower short- and long-term mortality rates in patients with TTS and cardiogenic shock [7]. Impella, a device that mechanically pumps blood from the LV into the aorta, has emerged as a promising option for maintaining hemodynamics in TTS patients with cardiogenic shock due to pump failure. Napp et al. reported from the retrospective registry that 13 patients (81.3%) out of 16 TTS patients with cardiogenic shock supported by Impella survived to discharge, and all patients showed significant recovery of LV systolic function [15]. For patients who remain hemodynamically unstable despite Impella, venoarterial extracorporeal membrane oxygenation (VA-ECMO) may be necessary as a bridge to LV contractility recovery.
2. RV involvement (Fig. 3)

**Fig. 3:**
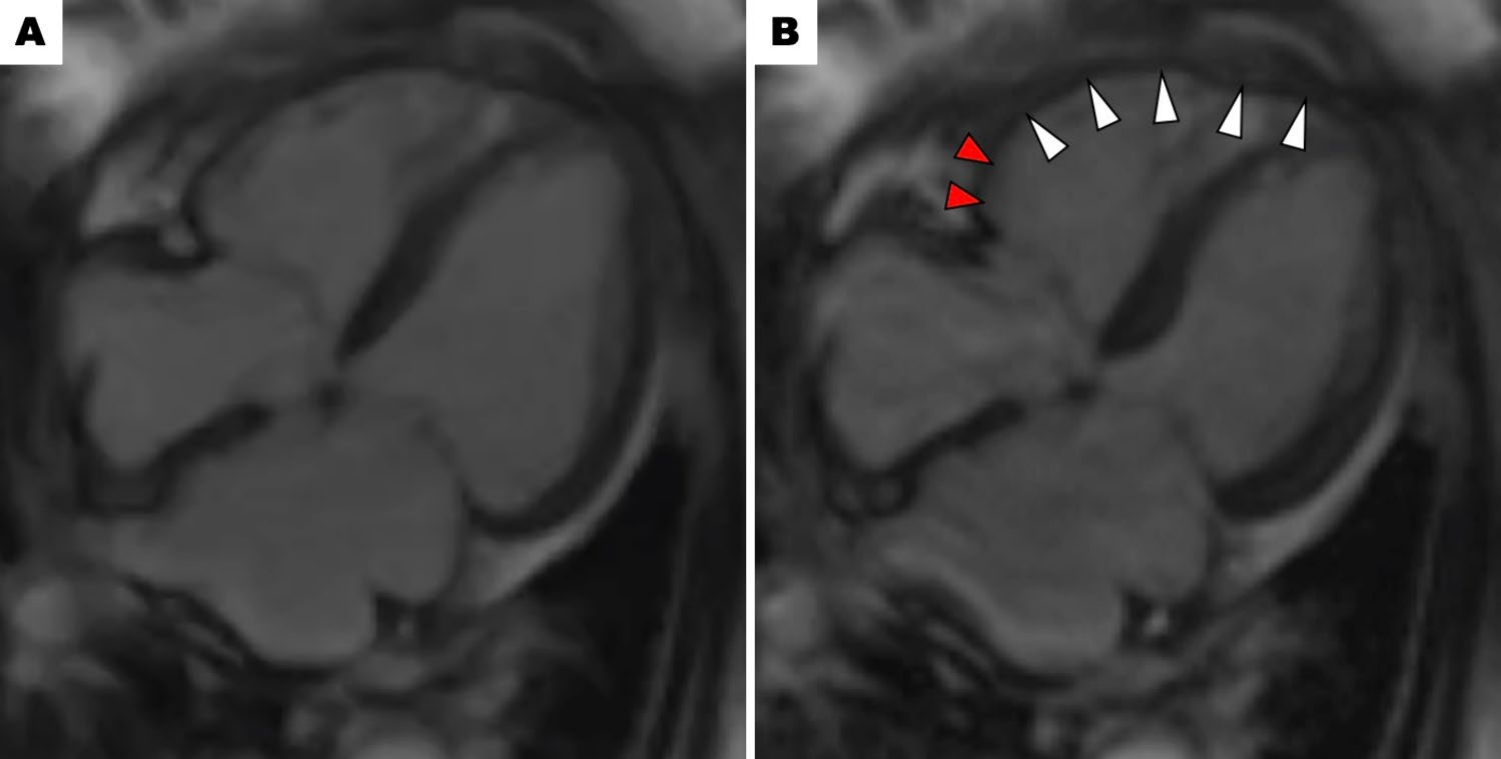
Right ventricular involvement. Cardiac magnetic resonance imaging demonstrates ballooning involving mid to apical segments (white arrows) and hyperkinetic basal segments (red arrows) of the right ventricle. Panels **A** (diastole) and **B** (systole)
  TTS is a disease that primarily presents with wall motion abnormalities in the LV, but in some cases, wall motion abnormalities are also apparent in the right ventricle (RV) [16]. Previous reports demonstrated that the prevalence of RV involvement is approximately 10–50% [17–21]. This variation may be due to the small number of cases, differences in the definition of RV wall motion abnormalities, and differences in imaging evaluation methods. Kagiyama et al. reported from an echocardiographic study that 18.6% of patients with TTS showed RV involvement, and patients with RV involvement more frequently experienced cardiogenic shock [18]. El-Battrawy et al. demonstrated that the incidence of RV involvement detected by echocardiography or cardiac magnetic resonance was 11%, and preceding physical trigger was an independent predictor of RV involvement. In addition, patients with RV involvement had a higher incidence of cardiogenic shock during hospitalization and a worse long-term outcome [19].
  If RV involvement is the primary cause or an exacerbating factor of cardiogenic shock in TTS, fluid administration is the first-line treatment. Agents that reduce preload, like diuretics and nitroglycerin, are not recommended as they can further compromise hemodynamics. If fluid administration is ineffective for improving hemodynamic status, additional use of inotropic agents might be considered. Non-catecholaminergic inotropic agents like levosimendan and milrinone are preferred in the treatment of TTS, but such agents should be used with caution in patients with RV involvement because of their potential vasodilatory effects that could worsen RV preload. In cases of persistent hypotension despite improved contractility, vasopressors like norepinephrine or vasopressin may be necessary.
3. LVOTO (Fig. 4)

**Fig. 4:**
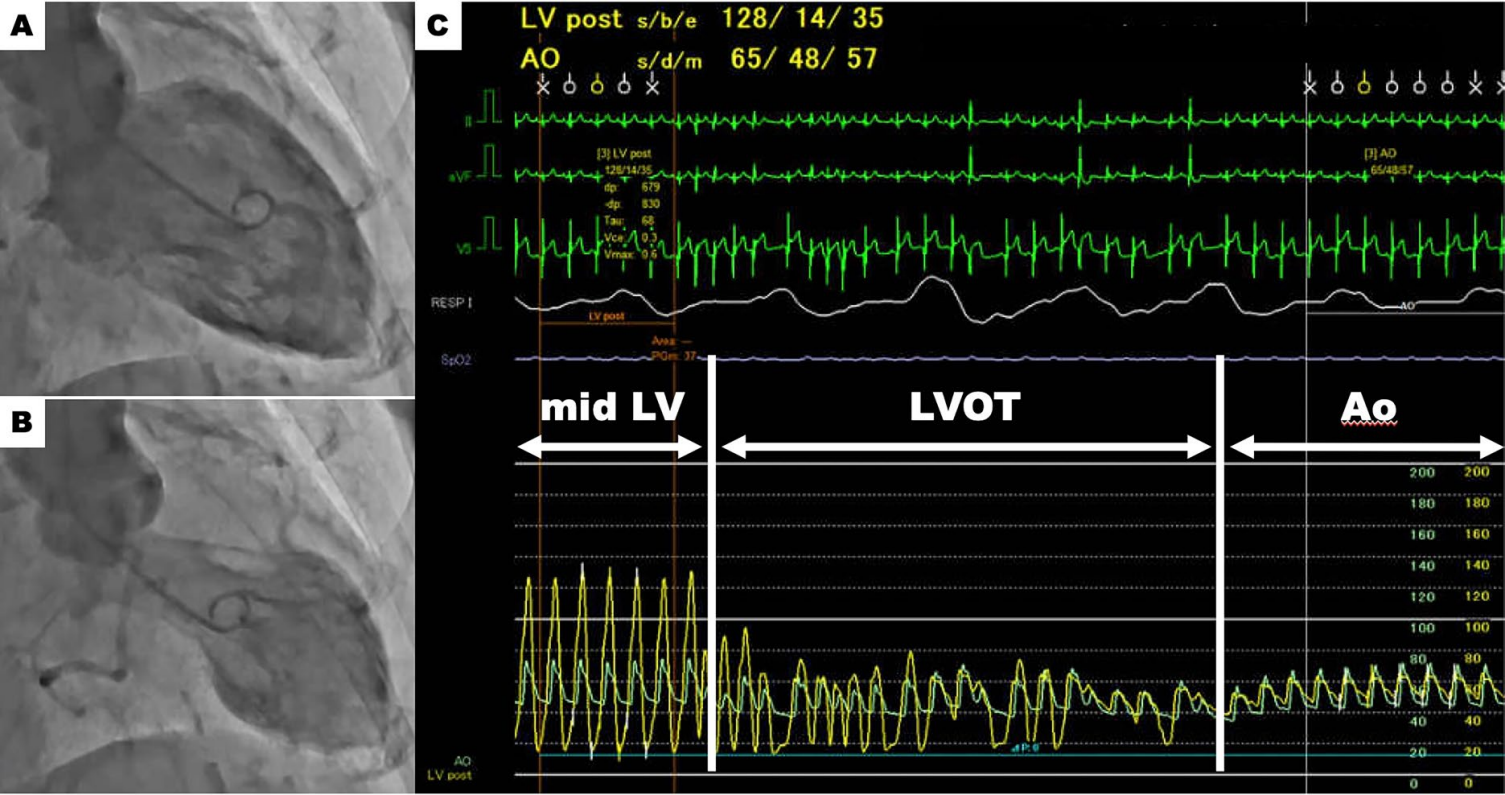
Left ventricular outflow tract obstruction. Left ventriculography demonstrates apical ballooning. Panels **A** (diastole) and **B** (systole). Pressure recording using a pigtail catheter slowly withdrawn from the left ventricle to the aorta reveals a remarkable peak-to-peak pressure gradient of 63 mmHg (Panel **C**). This finding suggests a significant left ventricular outflow tract obstruction. *Ao* aorta; *LV* left ventricle; *LVOT* left ventricular outflow tract
  In TTS with cardiogenic shock, identification of LVOTO is crucial in determining the course of treatment [22]. The presence of an intraventricular systolic pressure gradient should be screened noninvasively by TTE or invasively during left ventriculography, with slow pullback of pigtail catheter from the LV to the aorta. The rate of LVOTO in TTS is reported to range between 7% and 33% [4, 23–26]. Citro et al. reported that LVOTO was associated with adverse in-hospital events [25]. Although the mechanism of LVOTO in TTS has not been fully elucidated, the paradoxical hypercontraction of the LV base and myocardial stunning beyond the mid LV, induced by a rapid increase in plasma catecholamine concentration, may be the main cause of the LV intraventricular pressure disparity. Additionally, small left ventricular cavities and pre-existing asymmetric hypertrophy may increase the risk of LVOTO development.
  If LVOTO is confirmed in patients with TTS and cardiogenic shock, the first-line therapy is fluid administration under close hemodynamic monitoring. In such cases, positive inotropic agents (both catecholamine and non-catecholamine) should not be used, because positive inotropic agents may enhance basal hypercontractility and worsen LVOTO. Also diuretic treatment is not recommended because LVOTO may be worsen via reduced preload. On the other hand, short-acting intravenous β-blockers (e.g., esmolol, landiolol) may decrease LVOT pressure gradient by reducing basal hypercontractility and improve hemodynamic status, while there are no established guidelines. Because the negative inotropic effects of beta-blockers may induce further hypotension, beta-blockers should be started cautiously at low doses and discontinued immediately if hemodynamic compromise is observed.
  If pharmacologic therapy fails to achieve hemodynamic stabilization, mechanical circulatory support may be necessary. IABP are contraindicated in LVOTO cases due to the risk of further obstruction. Impella, on the other hand, offers a valuable option. It bypasses the LVOT by directly drawing blood from the LV and discharging it into the aorta, potentially improving hemodynamics and serving as a bridge to LV contractility recovery.
4. Acute MR (Fig. 5)

**Fig. 5:**
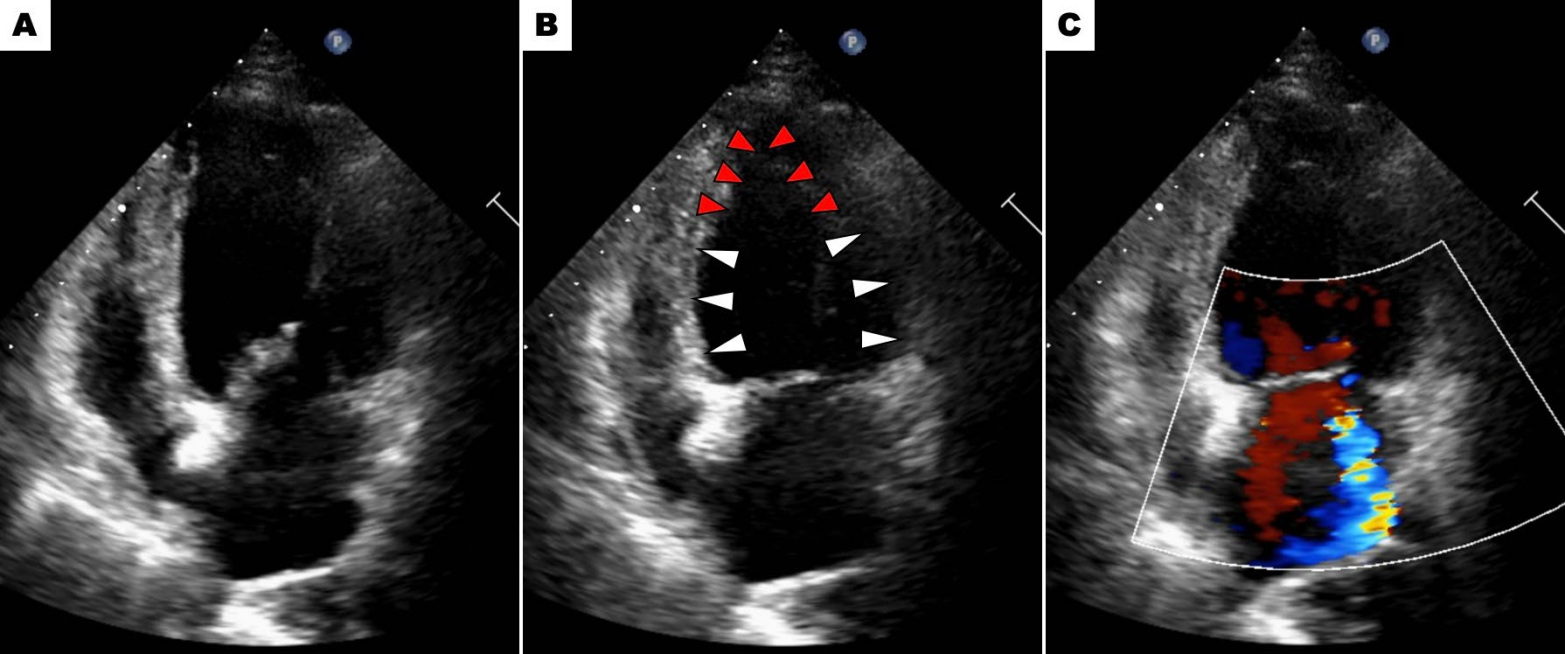
Acute mitral regurgitation. Echocardiography demonstrates circumferential ballooning involving basal to mid segments (white arrows) and hyperkinetic apical segments (red arrows) of the left ventricle (basal type). Panels **A** (diastole) and **B** (systole). Color Doppler shows severe mitral regurgitation due to mitral valve tethering (Panel **C**)
  Acute MR is a potential complication of TTS and can occasionally contribute to cardiogenic shock. The reported prevalence of significant acute MR (moderate or severe) in TTS detected by echocardiography or left ventriculography ranges from 15% to 25% [25, 27–30]. Citro et al. reported that acute MR is an independent risk factor for the composite endpoint of acute heart failure, cardiogenic shock, and in-hospital mortality, showing a stronger association compared to LVOT and RV involvement [25]. Izumo et al. reported that there are two main mechanisms for acute MR in TTS: (1) systolic anterior motion of the mitral valve (SAM) secondary to LVOTO, (2) mitral valve tethering due to papillary muscle displacement associated with extensive left ventricular wall motion abnormality and/or systolic dysfunction of papillary muscle [28]. The therapeutic approach for acute MR depends on its underlying mechanism, necessitating a comprehensive echocardiographic evaluation.
  If SAM is the main cause of acute MR, treatment aligns with the management of cardiogenic shock secondary to LVOTO. However, fluid administration requires caution due to the high frequency of pulmonary congestion in patients with acute MR. Administering short-acting β-blockers intravenously, like esmolol or landiolol, is often an effective strategy to resolve LVOTO and alleviating acute MR. Vasopressors (e.g., norepinephrine, vasopressin) may be necessary to maintain blood pressure if it drops further after beta-blocker administration.
  Treatment for acute MR mainly due to mitral valve tethering follows the approach for cardiogenic shock caused by primary pump failure. As mentioned earlier, the use of non-catecholaminergic inotropic agents is recommended to enhance cardiac contractility. If blood pressure drops due to the vasodilatory effects of these inotropic agents, vasopressors may be needed to maintain blood pressure.
  If medications fail to achieve hemodynamic improvement, Impella, a powerful mechanical assist device, can be used as a bridge to recovery regardless of the underlying cause of acute MR.

## Conclusion

Cardiogenic shock is a serious complication of TTS arising from various mechanisms, including pump failure, RV involvement, LVOTO, and acute MR. Early and accurate identification of the underlying mechanism using echocardiography is crucial for selecting the most appropriate treatment approach. By tailoring therapy to the specific pathophysiology, clinicians can optimize patient outcomes and improve prognosis in TTS patients with cardiogenic shock.
